# Deprescribing benzodiazepines and Z-drugs in community-dwelling adults: a scoping review

**DOI:** 10.1186/s40360-015-0019-8

**Published:** 2015-07-04

**Authors:** André S. Pollmann, Andrea L. Murphy, Joel C. Bergman, David M. Gardner

**Affiliations:** 1 grid.55602.340000000419368200Faculty of Medicine, Dalhousie University, Mail Box #259, 5849 University Avenue, Room C-125, PO Box 15000, Halifax, NS B3H 4R2 Canada; 2 grid.55602.340000000419368200College of Pharmacy and Department of Psychiatry, Dalhousie University, 5968 College St, PO Box 15000, Halifax, NS B3H 4R2 Canada; 3 grid.55602.340000000419368200Department of Psychiatry and College of Pharmacy, Dalhousie University, QEII HSC, AJLB 7517, 5909 Veterans’ Memorial Lane, Halifax, NS B3H 2E2 Canada

**Keywords:** Benzodiazepines, Z-drugs, Deprescribing, Clinical pharmacology, Behaviour change wheel, Scoping review

## Abstract

**Background:**

Long-term sedative use is prevalent and associated with significant morbidity, including adverse events such as falls, cognitive impairment, and sedation. The development of dependence can pose significant challenges when discontinuation is attempted as withdrawal symptoms often develop. We conducted a scoping review to map and characterize the literature and determine opportunities for future research regarding deprescribing strategies for long-term benzodiazepine and Z-drug (zopiclone, zolpidem, and zaleplon) use in community-dwelling adults.

**Methods:**

We searched PubMed, Cochrane Central Register of Controlled Trials, EMBASE, PsycINFO, CINAHL, TRIP, and JBI Ovid databases and conducted a grey literature search. Articles discussing methods for deprescribing benzodiazepines or Z-drugs in community-dwelling adults were selected.

**Results:**

Following removal of duplicates, 2797 articles were reviewed for eligibility. Of these, 367 were retrieved for full-text assessment and 139 were subsequently included for review. Seventy-four (53 %) articles were original research, predominantly randomized controlled trials (n = 52 [37 %]), whereas 58 (42 %) were narrative reviews and seven (5 %) were guidelines. Amongst original studies, pharmacologic strategies were the most commonly studied intervention (n = 42 [57 %]). Additional deprescribing strategies included psychological therapies (n = 10 [14 %]), mixed interventions (n = 12 [16 %]), and others (n = 10 [14 %]). Behaviour change interventions were commonly combined and included enablement (n = 56 [76 %]), education (n = 36 [47 %]), and training (n = 29 [39 %]). Gradual dose reduction was frequently a component of studies, reviews, and guidelines, but methods varied widely.

**Conclusions:**

Approaches proposed for deprescribing benzodiazepines and Z-drugs are numerous and heterogeneous. Current research in this area using methods such as randomized trials and meta-analyses may too narrowly encompass potential strategies available to target this phenomenon. Realist synthesis methods would be well suited to understand the mechanisms by which deprescribing interventions work and why they fail.

**Electronic supplementary material:**

The online version of this article (doi:10.1186/s40360-015-0019-8) contains supplementary material, which is available to authorized users.

## Background

Benzodiazepines and similar sedative hypnotics, including zopiclone, zaleplon, and zolpidem (“Z-drugs”), are extensively prescribed medications in the community setting [1–7]. The annual incidence of long-term benzodiazepine use across North America and Europe is estimated to be between 0.4 % to 6 %, with higher rates of chronic use in patients older than 65 years [5, 8–10]. The prevalence of benzodiazepine use in adults aged 18 to 64 years has remained relatively stable over the past decade, suggesting potential issues with long-term use beyond what is normally indicated [3, 5, 11]. Recent data from Canada suggest important changes in prescribing practices.  New prescriptions for benzodiazepines are declining, especially in older adults, while Z-drug use has steadily increased [5]. These trends mirror similar findings from other international studies [8, 12–18].

While indicated only for short-term management of anxiety and insomnia, reasons for acute benzodiazepine and Z-drug therapy transforming into chronic use are complex. Several prescriber related factors are believed to influence this process. These factors may include the prescriber’s attitudes toward these medications and toward the ‘deserving’ patient, deficits in specialized knowledge about sedative prescribing, the clinical work environment, conflicting patient health priorities, and the prescribing practices of others involved in the patient’s care [19, 20]. The perceived or real inaccessibility to alternative treatment modalities may further encourage the renewal of benzodiazepine and Z-drug prescriptions in favor of initiating other interventions that are perceived as less effective [21]. Patient factors including disagreement with appropriateness of cessation, fears of symptom return, withdrawal experiences, and the impression of unsuitability of alternatives also act to promote continued use [22, 23]. Considering the highly varied contributing factors that lead to long-term benzodiazepine and Z-drug use, deprescribing strategies need to be flexible and acceptable to both patients and clinicians.

Deprescribing is the collaborative and supportive process of identifying, modifying, and discontinuing therapies that are no longer indicated or may be causing harm to patients [24, 25]. Research and clinical programs for deprescribing typically focus on elderly patients due to high rates of medication-related morbidity and mortality, such as falls, fractures, motor vehicle collisions, daytime sedation, and cognitive impairment [26–31]. However, stable prevalence of benzodiazepine use and increasing Z-drug use in adults will also require that best-practice deprescribing strategies in this population be identified.

Numerous pharmacologic and nonpharmacologic deprescribing strategies have been reported in the literature with significant heterogeneity in the range and scope of psychological therapies, pharmacotherapy substitution approaches, and gradual dose reduction (GDR) schedules. We conducted a scoping review to map and characterize the literature, identify potential research gaps, and determine opportunities for future systematic syntheses regarding strategies and behaviour change interventions for deprescribing benzodiazepines and Z-drugs in community-dwelling adults who are long-term users (i.e., eight weeks or longer).

## Methods

Scoping review methods are appropriate for our topic area given the complexity and heterogeneity of existing research [32]. The intention is to characterize and map the literature, identify research gaps, and prioritize targeted areas for future reviews and research [33]. We also aimed to explicate various interventions used in the literature and characterize them according to the Behaviour Change Wheel based on the work of Michie et al. [34]. We followed scoping review procedures slightly modified, but as outlined by Arskey and O’Malley [35] and further explicated by Levac et al. [32] and others [33, 36–39]. Our review was conducted in six iterative stages including developing the research question, identifying relevant articles, selecting articles, extracting data, collating results, and engaging stakeholders through consultation (e.g., presentations on the topic) (Additional file 1).

### Definitions and search strategies

As a research team we met and reached consensus on population, intervention, comparator, and outcome definitions (Additional file 1). We limited our target population to patients taking benzodiazepines and Z-drugs in the community or outpatient settings as individuals receiving care in inpatient, long-term care, or residential aged care facilities can differ systematically with respect to numerous factors. These factors include, but are not limited to, the context of the environment, frailty, nature and number of illnesses, and treatment goals. Long-term use was defined as regular use beyond an eight-week period. The target medications for this review included all benzodiazepines and Z-drugs, defined as “zopiclone”, “eszopiclone”, “zolpidem”, or “zaleplon”.

Due to the broad nature of scoping reviews, we did not limit our research question to a particular type of intervention or comparator and included studies investigating pharmacologic, psychological, and various mixed methods of discontinuing benzodiazepine or Z-drug therapy. We classified pharmacologic interventions as those adding additional drug therapy (non-benzodiazepine or Z-drug) to facilitate discontinuation of the sedative or mitigate withdrawal symptoms. Psychological interventions were those utilizing behavioural techniques, such as cognitive behavioural therapy (CBT), to reduce benzodiazepine or Z-drug use. We categorized studies as mixed interventions if they compared various pharmacologic, psychological, or other interventions with each other. GDR included employing a taper regimen or switching between sedatives to facilitate benzodiazepine or Z-drug withdrawal. The remaining intervention types not falling within these categories were classified as ‘other’ (i.e., letter or brief consultation).

We collaborated with a medical science librarian to develop search methods for each database and to identify key terms and relevant medical subject headings. Our searches were developed to model the PICO (Population, Intervention, Comparator, Outcome) format for clinical questions [40].We searched PubMed, EMBASE, PsycINFO, the Cochrane Central Register of Controlled Trials (CENTRAL), CINAHL, and JBI Ovid databases from inception to December 19, 2013. Systematic combinations of the medical subject headings “benzodiazepine”, “hypnotics and sedatives”, “substance withdrawal syndrome”, “dependency”, “sleep disorders”, and “anxiety disorders” were used together with the keywords “hypnotic”, “sedative”, “zopiclone”, “eszopiclone”, “zolpidem”, “zaleplon”, “withdraw*”, “deprescrib*”, “taper”, “stop”, and “discontinu*”. Search terms were translated as appropriate for each database.

To identify further references not captured in the published medical literature, we used relevant sections of the Canadian Agency for Drugs and Technologies in Health’s (CADTH) “Grey Matters: a practical search tool for evidence-based medicine” [41] to search 69 international grey literature sources from the earliest available date through January 30, 2014. We also searched Opengrey (SIGLE), Google Advanced, screening the first 100 results for relevance to our clinical question, and the Turning Research Into Practice (TRIP) database for clinical practice guidelines concerning deprescribing of benzodiazepines and Z-drugs. Additional articles potentially relevant to our objectives were identified through reviewing reference lists of articles captured in our initial searches and by engaging with experts and colleagues.

### Study selection

We used pre-defined inclusion criteria to select articles identified through the search strategy that were relevant to our study objectives. We included those studies that were published in English and investigated or discussed methods for discontinuing benzodiazepines and sedative hypnotics in community-dwelling individuals aged 18 years and older. Based on our pre-determined criteria, we did not include studies that exclusively investigated benzodiazepine and Z-drug use in patients with conditions other than anxiety or insomnia disorders. We excluded studies in animals, pediatric patients (<18 years old), and short-term users of benzodiazepines or Z-drugs (less than eight weeks). We did not include studies investigating non-clinical outcomes (e.g., electroencephalography and brain imaging studies), and articles not investigating benzodiazepine and Z-drug discontinuation as a primary focus. With the exception of case-reports, case-series, and commentaries, articles were not excluded based on methodology or publication type as we sought to identify trends across the wide spectrum of research and publications in the area [35]. Original investigations, research syntheses, guidelines, and narrative review articles were all eligible for inclusion in order to capture potential differences amongst these publications with respect to benzodiazepine and Z-drug deprescribing recommendations. Indicators of study quality were broadly assessed as a means to understand the nature of research methods used and reported, but study quality was not used as an inclusion criterion.

The list of article titles and abstracts resulting from the database and grey literature searches were scanned independently by two reviewers (AP and JB), who assigned a value of “include”, “exclude”, or “assess further” to each reference. After the initial screening phase, full-text articles were retrieved and independently assessed by AP and JB for inclusion in the review using forms for determining eligibility criteria. Disagreements between the two assessors were discussed, and a third author (AM) was consulted if agreement could not be reached.

### Collating, summarizing, and reporting results

The data abstraction tool was drafted and revised through meetings throughout the stages of the review (Additional file 1). The standardized form was designed to capture information about the year of publication, country of origin, study type, target medication investigated, types of interventions, GDR protocol if used, duration of intervention, and information about the participants. Behaviour change interventions used in original research trials were categorized according to the Behaviour Change Wheel as enablement (“increasing means/reducing barriers to increase capability or opportunity”), training (“imparting skills”), persuasion (“using communication to induce positive or negative feelings or stimulate action”), environmental restructuring (“changing the physical or social context”), modeling (“providing an example for people to aspire to”), education (“increasing knowledge or understanding”), incentivisation (“creating expectation of reward”), coercion (“creating expectation of punishment or cost”), and restriction (“using rules to reduce opportunity to engage in target behaviour”) [34]. Study methodology was characterized according to the Agency for Healthcare Research and Quality’s (AHRQ) “Assessing Risk of Bias and Confounding in Observational Studies of Interventions or Exposures: Further Development of the RTI Item Bank” [42] with the addition of systematic reviews and narrative review articles. We categorized general data regarding the direction of effect of endpoints related to benzodiazepine and Z-drug discontinuation as a formal evaluation of the effect size of specific interventions is beyond the objective of a scoping review.

Prior to beginning the abstractions, an abstraction meeting was held to outline the process and model how to characterize intervention functions to establish consistency among team members. Initial articles were abstracted in a group environment so that discussion could occur surrounding issues or uncertainties regarding intervention function categorization. Following the initial abstractions one investigator (AP) reviewed all article abstractions for consistency in terminology, accuracy, and comprehensiveness. The PRISMA checklist form is provided as Additional file 2.

## Results

### Search

**Fig. 1:**
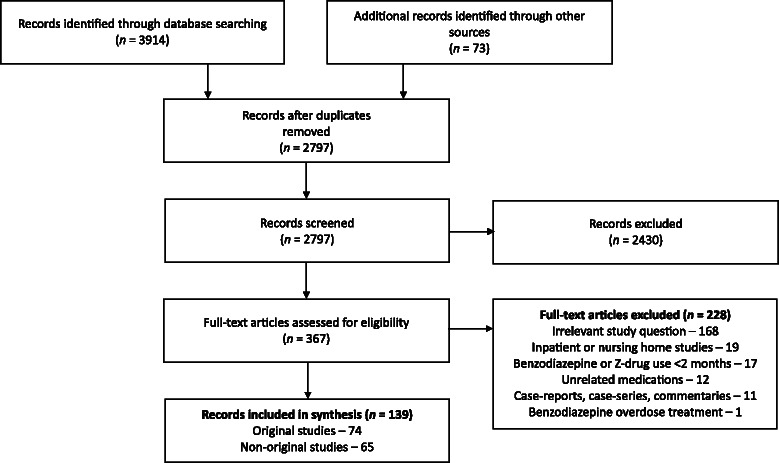
PRISMA flow diagram showing results of search and process of selecting articles for review

Our literature search yielded 2797 articles after duplicates were removed. Review of titles and abstracts led to retrieval of 367 full-text articles for assessment (Fig. 1). Of these, 74 original research studies and 65 review articles or guidelines were included (Additional file 3) [8, 43–180]. The absence of benzodiazepine or Z-drug discontinuation strategies as a major focus of the intervention or outcome was the most frequent reason for excluding articles.

### Description of articles

Deprescribing articles for benzodiazepines and Z-drugs were published between 1982 and 2014 with no apparent trend towards growing publications in this field over the past 30 years (Table 1). Research was primarily conducted in the United States (27 %) and United Kingdom (24 %). Articles were published in a variety of journals with scopes including psychiatry (n = 43 [31 %]), general/primary care (n = 37 [27 %]), pharmacology (n = 28 [20 %]), psychology and behavioural sciences (n = 11 [8 %]), and addiction (n = 11 [8 %]). Most original research studies were randomized controlled trials (RCTs) (n = 52 [37 %]), but nearly 50 % of all included articles were non-original research (Table 1). The majority of studies were relatively small, with less than 100 participants in total (n = 51 [69 %]). The setting of original research included primary care and outpatient clinics (n = 45 [61 %]), specialty clinics (n = 14 [19 %]), and university research units (n = 8 [11 %]), while seven studies did not report setting details (10 %).

**Table 1:** Characteristics of publications on benzodiazepine and Z-drug discontinuation in community dwelling adults

Characteristic	No.	% of 139 articles
Type of article		
*Original research*		
Randomized-controlled trial	52	37.4 %
Before-after study	8	5.8 %
Nonrandomized controlled trial	6	4.3 %
Systematic review/meta-analysis	3	2.2 %
Prospective cohort study	2	1.4 %
Non-comparative study	2	1.4 %
Retrospective cohort study	1	0.7 %
*Non-original research*		
Narrative review	58	41.7 %
Guideline	7	5.0 %
Country of origin		
United States	38	27.3 %
United Kingdom	33	23.7 %
Europe	24	17.3 %
Canada	20	14.4 %
Australia	13	9.4 %
Asia	2	1.4 %
Other	9	6.5 %
Year of publication		
<1985	4	2.9 %
1985–1994	50	36.0 %
1995–2004	40	28.8 %
2005–2015	45	32.4 %
Primary medical condition		
Mixed	73	52.5 %
Anxiety disorders	27	19.4 %
Insomnia	23	16.5 %
Panic disorders	16	11.5 %

Amongst research trials, most (n = 36 [49 %]) investigated patient populations taking benzodiazepines for multiple reasons. Insomnia or anxiety disorders were the primary diagnosis in 16 studies each (22 %). Fewer (n = 6 [8 %]) trials focused on patients with panic disorder. In contrast, 57 % (n = 37) of review articles examined mixed conditions, 11 % (n = 7) insomnia, 17 % (n = 11) anxiety disorders, and 15 % (n = 10) panic disorders. The distribution of studies reporting a mean age of 40 to 49 years, 50 to 59 years and 60 to 69 years, was 43 % (n = 32), 18 % (n = 13), and 19 % (n = 14), respectively. The duration of prior benzodiazepine or Z-drug use varied widely among studies and details concerning the length of therapy were not reported in 41 % (n = 30). The mean duration of benzodiazepine or Z-drug use was typically less than a decade (n = 30 [41 %]). The majority of original trials (n = 41 [55 %]) investigated the discontinuation of any benzodiazepine, whereas 16 % (n = 12) examined both benzodiazepine and Z-drug discontinuation. Only three (4 %) of 74 original studies exclusively examined strategies for stopping Z-drugs. The remaining studies enrolled patients only if they were taking specific benzodiazepines, most frequently alprazolam (n = 11), diazepam (n = 7), and lorazepam (n = 6).

The general direction of effect for the endpoint of discontinuation of benzodiazepine or Z-drug therapy was noted for each research trial, regardless of the type of intervention studied. Of original research studies, 41 % (n = 30) of studies demonstrated a negative (or non-significant) effect of the intervention being investigated. A positive effect was demonstrated in 47 % (n = 35) of studies, while 12 % (n = 9) of research studies did not provide sufficient data to clearly assess the direction of the effect (Additional file 3).

### Deprescribing strategies

#### Pharmacologic

Among original research studies, pharmacologic interventions were the most common types of interventions assessed for their impact on reducing benzodiazepine and Z-drug exposure (n = 42 [57 %]) (Table 2). Thirty-three studies investigated the addition of pharmacologic therapies to facilitate benzodiazepine or Z-drug discontinuation. Amongst these, buspirone was the most frequently studied therapy in RCTs (n = 4) and nonrandomized controlled trials (n = 3), with a total of 275 subjects. Melatonin was studied in five trials, of which four were RCTs with 244 subjects. Sixteen other additive pharmacologic agents were studied, which included beta-adrenergic receptor antagonists (n = 3), anti-seizure drugs such as carbamazepine, pregabalin, and valproate (n = 5), and antidepressants such as imipramine, paroxetine, and trazodone (n = 5). Other medications investigated were ondansetron (n = 1) and progesterone (n = 1).

**Table 2:** Benzodiazepine and Z-drug deprescribing strategies studied or discussed in publications

Strategies researched in original studies	No.	% of 74
Pharmacologic therapy	42	56.8 %
Psychological therapy	10	13.5 %
Mixed	12	16.2 %
Other	10	13.5 %
Strategies discussed in non-original articles	No.	% of 65
Pharmacologic therapy	24	36.9 %
Psychological therapy	30	46.2 %
GDR	60	92.3 %
Other	21	32.3 %

#### Gradual dose reduction

Original research investigations of pharmacologic, psychological, mixed, and other interventions frequently included GDR as a component of the discontinuation method (60 of 74 [80 %]). However, the types of GDR regimens employed varied dramatically among trials and 15 studies did not report details about their GDR methods. Stabilization (i.e., establishing a consistent daily dose) of benzodiazepine or Z-drug dose prior to the initiation of tapering was a strategy employed in 28 % of studies. As part of the GDR regimen, 31 (42 %) trials established flexible taper plans, which involved altering the rate of taper based on patient symptoms. The remaining trials established a uniform tapering regimen that was applied to all subjects. In 13 trials (18 %), participants were switched to another agent, most frequently diazepam (n = 10), but also Z-drugs including zopiclone (n = 2) and zolpidem (n = 1). The time frame over which GDR was conducted ranged from one to more than 16 weeks with the most common (n = 11) being four weeks (median 6 weeks, interquartile range 4–8 weeks). Twenty studies employing GDR did not report the time frame of their taper regimen. The most common taper rate among studies (n = 16) was decreasing the original dose by 25 % weekly (i.e., 75 % of original dose for one week, then 50 % of original dose for one week, then 25 % of original dose for one week, then stop). Seven studies outlined a slower approach, decreasing the dose by 25 % every two to four weeks. Shorter tapers were also reported in seven studies, with the dose reduced by half for one to two weeks before the benzodiazepine or Z-drug was discontinued.

The majority of review articles and clinical practice guidelines (60 of 65 [92 %]) recommended GDR as part of a discontinuation strategy (Table 2). Recommendations concerning GDR strategy varied widely but a flexible approach and substitution of a long-acting benzodiazepine were frequent suggestions.

#### Psychological therapies

Psychological therapies to facilitate discontinuation of benzodiazepines and Z-drugs were studied in 10 trials (14 %), with 60 % of these trials utilizing CBT (Table 2). Other strategies employed via both group and individualized therapies included anxiety management, stress management, and psychotherapy. Nine of these 10 trials were RCTs with a total of 408 patients.

#### Mixed and other interventions

Mixed interventions comparing various pharmacologic, psychological, or other interventions were the focus of 12 (16 %) studies (Table 2). Five of these 12 trials compared more than two types of interventions. Common interventions included psychological therapy (most frequently CBT), GDR alone, and usual care. Other interventions that were investigated in 14 % of original research studies included interventions such as sending a letter or detailed information to patients and brief counseling by clinicians.

### Intervention functions

Six of the nine intervention types described by Michie et al., [34] were used in the research studies included. The majority of original studies included a single intervention function (n = 39 [53 %]), while the remainder combined two (n = 12 [16 %]), three (n = 21 [28 %]), or four (n = 2 [3 %]) distinct functions (Additional file 3). The most common method employed was enablement, which was used in 76 % (n = 56) of studies. The majority of studies using enablement techniques attempted to achieve benzodiazepine or Z-drug cessation by testing the effect of GDR, additional pharmacotherapy, or psychological therapies. Education and training were also frequent elements assessed by research studies, being a component of 47 % (n = 35) and 38 % (n = 28) of studies, respectively. A total of 11 studies investigated persuasion interventions (15 %), which frequently involved the physician sending a letter to patients to explain the harms of benzodiazepines or Z-drugs and encouraging discontinuation. Three trials tested environmental restructuring techniques to facilitate discontinuation. In addition to other strategies, one trial evaluated modeling as a component of a patient information package [8].

## Discussion

We identified 139 articles for deprescribing benzodiazepines and Z-drugs in community-dwelling adults that included a range of different strategies and behaviour change interventions. This is in contrast to recent meta-analyses that included 32 studies or fewer [117, 136, 138]. While meta-analyses of data can offer valuable answers to specific research and clinical questions, the strict inclusion criteria based on study methodology and quality can limit the amount of information they provide on the research area as a whole. Research and publications that would be characterized as lower levels of evidence in hierarchies [181–183] (e.g., non-randomized trials, guidelines, narrative reviews) can be captured in scoping reviews, which is important in many clinical questions given the kinds of evidence that inform clinicians and patients in decision-making [184]. In our scoping review, we included systematic reviews, individual RCTs, practice guidelines, reviews, and other study designs to characterize the literature, identify research gaps and future research priorities, and determine opportunities for future systematic syntheses regarding deprescribing strategies. This more inclusive approach reflects the sources of information utilized by clinicians, especially for therapeutic decisions that require individualized and flexible care plans, such as benzodiazepine and Z-drug discontinuation [185].

Pharmacologic interventions have been the primary discontinuation strategy reported on in the majority of studies within previous meta-analyses [117, 136, 138]. Likewise, our scoping review found that the addition of pharmacologic agents to facilitate discontinuation has been the most commonly studied type of intervention in RCTs (31 trials, totaling 2273 patients). This method of discontinuation may be counterintuitive to both prescribers and patients as risks for different adverse events and increased costs are inherent within this approach. Despite the majority of trials studying this method, non-original review articles and guidelines included in our scoping review did not discuss this approach as frequently. This is especially important to consider given the large degree of variability and tensions that can exist with the use of different forms of evidence in clinical decision-making [186–188]. Depending on the practitioner, guidelines and narrative reviews may be significantly influential in decision-making. Patients will also inherently use various forms of information about medications in their decision-making, much of which will not necessarily include information from clinical trials but that is readily accessible on the internet [189].

Our review revealed that benzodiazepine and Z-drug deprescribing interventions are numerous, largely heterogeneous, and poorly described. The pace of publication annually remained stable, indicating maintained interest in this field. Estimates of effect size direction, while not attributable to a specific intervention or intervention type, were mixed with 47 % of trials being positive, 41 % negative, and 12 % undetermined, suggesting a lack of clarity regarding how to best deprescribe benzodiazepines and Z-drugs. Replication of strategies in the clinical practice setting with fidelity to interventions that were studied is nearly impossible owing to the large number of approaches examined and the lack of details provided in some reports. Guidelines such as the Template for Intervention Description and Replication (TIDieR) [190] and the checklist by the Workgroup for Intervention Development and Evaluation Research (WIDER) [191] should be used to describe interventions in sufficient detail to allow for their replication not only for subsequent research but in clinical practice. It would also allow investigators to better develop implementation strategies for various interventions in a range of settings using implementation frameworks [192].

Limited theoretical underpinnings of interventions combined with the significant heterogeneity and complexity of strategies as found in our review, presents challenges in helping to understand and explain the mechanisms by which interventions work, why they work, for whom, and in which contexts. Furthermore, with rising use of Z-drugs and other psychotropics for insomnia (e.g., quetiapine) [193] we need to understand whether strategies that work for benzodiazepines are in fact those that work for discontinuing other types of hypnotics. We recommend that a realist synthesis [194] approach be used in future syntheses given the complexity of these issues and the need to better understand the mechanisms by which deprescribing interventions for benzodiazepines and Z-drugs work and why they fail [195].

The generalizability of results from available studies is problematic due to the risk of sample distortion bias and selection bias affecting the findings. Across several studies, subjects participated because they were already motivated to stop their benzodiazepine or Z-drug. For many existing users, there is significant reluctance or refusal to discontinue benzodiazepines and Z-drugs when given the opportunity [22, 196, 197]. Additionally, long-term users of benzodiazepines and Z-drugs may have comorbidities that would exclude them from participating in many deprescribing clinical trials. The goals of care in patients with ongoing comorbidities may be quite different and benzodiazepine and Z-drug deprescribing may not be an immediate priority. Participants in trials were often of younger age (i.e., 40 to 50 years), which does not match with the older population targeted by deprescribing initiatives that aim to reduce harm. Research in the area of deprescribing benzodiazepines and Z-drugs should focus on older people with a wide range of comorbidities.

To date, the outcome of interest in benzodiazepine and Z-drug deprescribing research has largely been whether or not treatment was successfully stopped. Clinical outcomes such as impact on reducing falls, fractures, quality of life, and mortality have been evaluated less frequently [108, 198]. Future research should determine the specific harms associated with long term sedative use, especially in vulnerable groups (e.g., frail adults, patients with multiple comorbidities), and aim to identify which patients benefit from benzodiazepine and Z-drug discontinuation in terms of quality of life, morbidity, and mortality.

There are limitations to this review. First, our search may not have been exhaustive, despite the search of multiple databases and grey literature sources. The lack of standardized medical subject headings in the developing area of deprescribing may have partially contributed to this. Second, we did not extract specific information about interventions studied and reviewed within the literature to determine which strategies are optimal for facilitating benzodiazepine and Z-drug discontinuation, as this is not the intended purpose of a scoping review. Third, although a predefined abstraction tool and classification scheme can minimize subjectivity among abstractors, there may be variability among investigators.

## Conclusions

Long-term and inappropriate use of benzodiazepines and Z-drugs remains a problem in community dwelling adults. Numerous pharmacologic, psychological, and other interventions have been used to support long-term benzodiazepine and Z-drug discontinuation, yet strategies are diverse and often poorly reported in sufficient detail to allow replication in future research or clinical practice. Using scoping review methods for this complex problem allowed for inclusion of a greater breadth of literature and the freedom to characterize and identify existing gaps in research, whereas traditional syntheses methods restrict clinicians to mostly RCT data of pharmacologic augmentation of GDR. Our results indicate that the current research in this area using methods such as RCTs and meta-analysis may too narrowly encompass the potential strategies available to target this phenomenon. Future studies in this area should describe interventions in sufficient detail, including information on various behaviour change techniques, to allow for their replication in research and clinical practice. This process could be facilitated by the use of standardized reporting guidelines and various checklists that currently exist [190, 191]. More research regarding the impact of deprescribing strategies on patient-centered outcomes in real-word settings is required. Realist synthesis methods would be well suited to understand the mechanisms by which deprescribing interventions for benzodiazepines and Z-drugs work and why they fail.

## Additional files

Additional file 1: Table S1. Research stages and methods for scoping review. *Summarizes the methods used for this scoping review, outlining the various stages and contributions of each author.*

Additional file 2: Table S2. PRISMA checklist.
Additional file 3: Table S3. Description of articles included in review. *This table displays information about the studies selected for inclusion in the review, including author, date of publication, study design, discontinuation strategies, age of participants studied, medication discontinued, key study outcomes, and Behaviour Change Wheel intervention functions employed.*

## Abbreviations

CBT: Cognitive behavioural therapy
GDR: Gradual dose reduction
RCT: Randomized controlled trial
