# Supplementary material for: Deprescribing benzodiazepines and Z-drugs in community-dwelling adults: a scoping review
Source: BMC Pharmacol Toxicol. 2015 Jul 4;16:19. doi: 10.1186/s40360-015-0019-8 (PMC4491204; doi:10.1186/s40360-015-0019-8)
Supplement: Additional file 1: Table S1. — Research stages and methods for scoping review. Summarizes the methods used for this scoping review, outlining the various stages and contributions of each author. [file 40360_2015_19_MOESM1_ESM.doc]

**Additional file 1: Table S1. Research stages and methods for scoping review.**

| **Stage** | **Purpose** | **Activities** | **Team Members Responsible** |
| --- | --- | --- | --- |
| 1 | Identifying Research Question | We held weekly meetings to discuss definitions and objectives of the review, identify key search terms, and potential literature sources to search. Preliminary categories for the data extraction tool were outlined. Our research question was structured. | AP, AM, DG |
| 2 | Identifying Relevant Studies | Preliminary study inclusion and exclusion criteria were developed and revised iteratively as needed. Database and grey literature searches were conducted. Search details were tracked and recorded and the results were organized using a reference database established in RefWorks. | AP, AM |
| 3 | Study Selection | Titles and abstracts were reviewed independently using pre-defined inclusion and exclusion criteria. Subsequently, full-text articles potentially relevant to our objectives where reviewed in full by AP and JB, with disagreements settled by consensus and consultation with AM. Final decisions and reasons for exclusion were recorded in an Excel spreadsheet. | AP, AM, JB |
| 4 | Data Extraction | The penultimate data extraction tool was finalized after a group-coding meeting. The first abstraction meeting was conducted in a group setting to further establish codes and processes. Each member of the coding team (n=7) abstracted at least five papers in the group setting, prior to completing the remaining abstractions independently. Following this AP reviewed all abstractions for completeness and accuracy. | AP, AM, JB, DG, additional supporting team members |
| 5 | Collating, Summarizing, and Reporting Results | This stage was broken down into steps including conducting analysis, reporting results, and interpreting the findings. A directed approach to content analysis was used to categorize textual data into themes. Following coding of all studies we identified prominent themes and subthemes. This strategy of deductive and inductive coding helped to identify important concepts and patterns in the data as well as trends and gaps in the evidence. Subsequently, the manuscript was drafted. | AP, AM, JB, DG |
| 6 | Stakeholder Engagement | We engaged stakeholders through presentations on the topic area. Knowledge translation and dissemination will occur. The results will be used in various ongoing research and clinical pursuits but will also be translated into findings to policy makers. | AP, AM, DG |
