# Supplementary material for: Deprescribing benzodiazepines and Z-drugs in community-dwelling adults: a scoping review
Source: BMC Pharmacol Toxicol. 2015 Jul 4;16:19. doi: 10.1186/s40360-015-0019-8 (PMC4491204; doi:10.1186/s40360-015-0019-8)
Supplement: Additional file 3: Table S3. — Description of articles included in review. This table displays information about the studies selected for inclusion in the review, including author, date of publication, study design, discontinuation strategies, age of participants studied, medication discontinued, key study outcomes, and Behaviour Change Wheel intervention functions employed. [file 40360_2015_19_MOESM3_ESM.doc]

**Additional file 3: Table S3.** Description of articles included in review.

Abbreviations: CBT, cognitive-behavioural therapy; GDR, gradual dose reduction; NHS, National Health Service; NOUGG, National Opioid Use Guideline Group; RCT, randomized controlled trial.

| **Ref** | **Source** | **Year** | **Article Type** | **Sample Size** | **Mean Age** | **Deprescribing Interventions Studied or Discussed** | **Medication Discontinued** | **Intervention Functions** | **Key Study Outcomes** | **Direction of Effect** |
| --- | --- | --- | --- | --- | --- | --- | --- | --- | --- | --- |
| 43 | Abelson et al. | 1993 | Non-comparative study | 18 | 30 | Alprazolam discontinuation and panic disorder relapse | Alprazolam | Enablement | Panic disorder relapse, anxiety severity, alprazolam discontinuation rates | Unclear |
| 44 | Ahmed et al. | 2008 | Narrative review | NA | NA | Discontinue "as needed benzodiazepine use", control of underlying condition (anxiety) prior to starting taper | NA | NA | NA | NA |
| 45 | Allain et al. | 1998 | RCT | 84 | 54 | Zolpidem vs. placebo | Triazolam | Enablement | Withdrawal symptoms, sleep quality measures | Positive |
| 46 | Ashton | 1994 | Narrative review | NA | NA | GDR, written GDR schedule, long-acting benzodiazepine substitution, patient education, psychological support | NA | NA | NA | NA |
| 47 | Ashton et al. | 1990 | RCT | 23 | 42 | Buspirone vs. placebo | Diazepam | Enablement | Withdrawal symptoms, adjuvant medications taken, drop-out rates, psychiatric rating scales (anxiety, depression) | Negative |
| 48 | Baillargeon et al. | 2003 | RCT | 65 | 67 | CBT and GDR vs. GDR alone | Any benzodiazepine | Training, education | Benzodiazepine discontinuation or dose reduction | Positive |
| 49 | Ballenger | 1991 | Narrative review | NA | NA | GDR | NA | NA | NA | NA |
| 50 | Ballenger et al. | 1993 | Narrative review | NA | NA | GDR, attempt to withdraw at a low-stress time in patient's life | NA | NA | NA | NA |
| 51 | Bartholomew | 1990 | Narrative review | NA | NA | GDR, long-acting benzodiazepine substitution, pharmacological therapies, psychological therapies | NA | NA | NA | NA |
| 52 | Bashir et al. | 1994 | RCT | 109 | 62 | Advice from physician vs. no intervention | Any benzodiazepine | Education | Benzodiazepine discontinuation or dose reduction | Positive |
| 53 | Bélanger et al. | 2009 | Narrative review | NA | NA | Patient education, GDR, CBT | NA | NA | NA | NA |
| 54 | Belleville et al. | 2007 | RCT | 53 | 55 | GDR and self-help CBT program vs. GDR alone | Any benzodiazepine or Z-drug | Enablement, training, education | Benzodiazepine or Z-drug use, sleep parameters, adherence to CBT intervention | Negative |
| 55 | Blais et al. | 1990 | Narrative review | NA | NA | Long-acting benzodiazepine substitution, systematic planning of withdrawal, GDR, frequent follow-up, psychological therapies | NA | NA | NA | NA |
| 56 | Bobes et al. | 2012 | Before-after study | 282 | 41 | Pregabalin | Any benzodiazepine | Enablement | Withdrawal symptoms, benzodiazepine use, pregabalin tolerability, anxiety symptoms | Unclear |
| 57 | Burrows | 1990 | Narrative review | NA | NA | GDR | NA | NA | NA | NA |
| 58 | Burrows et al. | 1990 | Narrative review | NA | NA | GDR | NA | NA | NA | NA |
| 59 | Cantopher et al. | 1990 | RCT | 31 | 46 | GDR vs. abrupt benzodiazepine discontinuation and propranolol | Diazepam | Enablement | Withdrawal symptoms, drop-out rates, psychiatric rating scales (anxiety, depression), benzodiazepine discontinuation | Negative |
| **Ref** | **Source** | **Year** | **Article type** | **Sample size** | **Mean age** | **Deprescribing interventions studied or discussed** | **Medication discontinued** | **Intervention functions** | **Key study outcomes** | **Direction of effect** |
| 60 | Chang | 2005 | Narrative review | NA | NA | GDR, long-acting benzodiazepine substitution | NA | NA | NA | NA |
| 61 | Choy | 2007 | Narrative review | NA | NA | Patient education, follow-up, long-acting benzodiazepine substitution, GDR | NA | NA | NA | NA |
| 62 | Chung et al. | 1999 | Before-after study | 109 | 56 | Brief education and GDR pamphlet | Any benzodiazepine | Education, training | Benzodiazepine discontinuation or dose reduction, anxiety symptoms | Positive |
| 63 | Cormack et al. | 1983 | Prospective cohort study | 50 | 52 | Letter encouraging benzodiazepine GDR and psychological treatment vs. letter alone | Any benzodiazepine | Training, enablement | Benzodiazepine discontinuation or dose reduction, anxiety symptoms | Negative |
| 64 | Cormack et al. | 1994 | Nonrandomized controlled trial | 209 | 69 | Letter alone vs. multiple letters vs. control | Any benzodiazepine or Z-drug | Education | Benzodiazepine discontinuation or dose reduction | Positive |
| 65 | Crouch et al. | 1988 | Prospective cohort study | 44 | 41 | Psychological group therapy, GDR, and propranolol/placebo/nothing vs. routine care | Any benzodiazepine | Education, training, enablement | Anxiety symptoms, drop out rates, self reported benzodiazepine use | Positive |
| 66 | Davidson | 1990 | Narrative review | NA | NA | GDR | NA | NA | NA | NA |
| 67 | Dell'osso et al. | 2013 | Narrative review | NA | NA | Minimal interventions, GDR, long-acting benzodiazepine substitution | NA | NA | NA | NA |
| 68 | Department of Health and Children, Ireland | 2002 | Guideline | NA | NA | GDR, follow-up, long-acting benzodiazepine substitution, pharmacological therapies | NA | NA | NA | NA |
| 69 | Department of Health, United Kingdom | 2007 | Guideline | NA | NA | Long-acting benzodiazepine substitution, GDR, flexibility, psychological interventions | NA | NA | NA | NA |
| 70 | Drake | 1991 | Before-after study | 44 | 56 | Planned withdrawal protocol | Temazepam | Enablement | Sleep and mood assessments, success of GDR, adverse events, compliance | Positive |
| 71 | Dubovsky | 1990 | Narrative review | NA | NA | GDR, follow-up, long-acting benzodiazepine substitution | NA | NA | NA | NA |
| 72 | Dupont | 1990 | Narrative review | NA | NA | Patient education, GDR, pharmacological therapies to suppress withdrawal | NA | NA | NA | NA |
| 73 | Dupont | 1990 | Narrative review | NA | NA | GDR, long-acting benzodiazepine substitution, pharmacological therapies to suppress withdrawal | NA | NA | NA | NA |
| 74 | El-Guealy et al. | 2010 | Narrative review | NA | NA | GDR, long-acting benzodiazepine substitution, pharmacological therapies, CBT | NA | NA | NA | NA |
| 75 | Elsesser et al. | 1996 | RCT | 38 | 59 | Complaints management training and GDR vs. anxiety management training and GDR | Any benzodiazepine | Education, training, enablement | Benzodiazepine discontinuation or dose reduction, psychiatric rating scales (anxiety, depression), withdrawal symptoms | Positive |
| 76 | Federation of Texas Psychiatry | 2002 | Guideline | NA | NA | Long-acting benzodiazepine substitution | NA | NA | NA | NA |
| **Ref** | **Source** | **Year** | **Article type** | **Sample size** | **Mean age** | **Deprescribing interventions studied or discussed** | **Medication discontinued** | **Intervention functions** | **Key study outcomes** | **Direction of effect** |
| 77 | Fraser et al. | 1990 | RCT | 30 | 58 | No help vs. GP help vs. psychology help | Any benzodiazepine | Education, training, enablement | Number of benzodiazepine prescriptions received, anxiety symptoms | Negative |
| 78 | Fyer et al. | 1987 | Before-after study | 18 | 33 | Structured GDR protocol | Alprazolam | Enablement | Anxiety and phobia severity, benzodiazepine use, withdrawal symptoms | Negative |
| 79 | Garfinkel et al. | 1999 | RCT | 34 | 68 | Melatonin and GDR vs. placebo and GDR | Any benzodiazepine | Enablement | Sleep quality, benzodiazepine use | Positive |
| 80 | Gilhooly et al. | 1998 | RCT | 91 | Not specified | Replacement with alternate benzodiazepine vs. replacement with antihistamine or chloral hydrate | Temazepam | Training, enablement | Patient satisfaction with replacement, benzodiazepine use status | Negative |
| 81 | GlaxoSmithKleine | 2005 | RCT | 54 | 49 | Paroxetine vs. placebo | Any benzodiazepine | Enablement | Benzodiazepine discontinuation at 12 weeks | Positive |
| 82 | Gosselin et al. | 2006 | RCT | 61 | 50 | CBT and GDR vs. nonspecific psychological treatment and GDR | Any benzodiazepine | Training, education, enablement | Benzodiazepine discontinuation, compliance with taper, anxiety symptoms, withdrawal symptoms | Positive |
| 83 | Hadley et al. | 2012 | RCT | 106 | 42 | Pregabalin and GDR vs. placebo and GDR | Any benzodiazepine | Enablement | Benzodiazepine use status, anxiety symptoms, withdrawal symptoms | Negative |
| 84 | Hallstrom et al. | 1988 | RCT | 44 | 41 | CBT and propranolol/placebo/no treatment vs. group therapy and propranolol/placebo/no treatment | Any benzodiazepine | Enablement, training | Benzodiazepine discontinuation or dose reduction | Unclear |
| 85 | Harangozo et al. | 1991 | Narrative review | NA | NA | GDR, long-acting benzodiazepine substitution, pharmacological therapies, psychotherapy | NA | NA | NA | NA |
| 86 | Hayward et al. | 1989 | Narrative review | NA | NA | Pharmacological therapies, GDR, psychological therapies | NA | NA | NA | NA |
| 87 | Heather et al. | 2004 | RCT | 284 | 69 | Short consultation vs. letter vs. usual care | Any benzodiazepine | Education | Benzodiazepine use status | Positive |
| 88 | Higgitt et al. | 1985 | Narrative review | NA | NA | GDR, flexibility, pharmacological therapies, follow-up, social support | NA | NA | NA | NA |
| 89 | Hofmann et al. | 1999 | Narrative review | NA | NA | Panic disorder control, GDR | NA | NA | NA | NA |
| 90 | Holm et al. | 2000 | Narrative review | NA | NA | Zolpidem and zopiclone discontinuation, withdrawal symptoms | NA | NA | NA | NA |
| 91 | Hopkins et al. | 1982 | Before-after study | 78 | 60 | Benzodiazepine withdrawal program | Any benzodiazepine | Enablement | Benzodiazepine discontinuation and use, withdrawal symptoms, illness symptoms | Positive |
| 92 | Huston | 1992 | Narrative review | NA | NA | Patient education, GDR, follow-up, pharmacological therapy, reassurance | NA | NA | NA | NA |
| 93 | Jensen et al. | 2014 | Narrative review | NA | NA | Education, stop at low stress time, GDR, long-acting benzodiazepine substitution, CBT | NA | NA | NA | NA |
| 94 | Keck et al. | 1992 | Narrative review | NA | NA | Pharmacologic therapies | NA | NA | NA | NA |
| **Ref** | **Source** | **Year** | **Article type** | **Sample size** | **Mean age** | **Deprescribing interventions studied or discussed** | **Medication discontinued** | **Intervention functions** | **Key study outcomes** | **Direction of effect** |
| 95 | Kenny et al. | 2009 | Guideline | NA | NA | GDR, planned approach, follow-up, long-acting benzodiazepine substitution, pharmacological therapies, CBT | NA | NA | NA | NA |
| 96 | Klein | 2002 | Narrative review | NA | NA | Long-acting benzodiazepine substitution, education, preparation, communication | NA | NA | NA | NA |
| 97 | Klein et al. | 1994 | RCT | 71 | 36 | Carbamazepine and GDR vs. placebo and GDR | Alprazolam | Enablement | Ability to complete four benzodiazepine-free weeks post GDR | Positive |
| 98 | Kunz et al. | 2012 | Retrospective cohort study | 512 | 63 | Melatonin | Any benzodiazepine or Z-drug | Enablement | Benzodiazepine or Z-drug use status after 3 months | Positive |
| 99 | Lader | 2011 | Narrative review | NA | NA | GDR, long-acting benzodiazepine substitution, CBT, pharmacological therapies | NA | NA | NA | NA |
| 100 | Lader | 1987 | Narrative review | NA | NA | Psychological therapies, pharmacological therapies, GDR, long-acting benzodiazepine substitution | NA | NA | NA | NA |
| 101 | Lader et al. | 1993 | RCT | 25 | 45 | Alpidem and GDR vs. placebo and GDR | Any benzodiazepine | Enablement | Completion rates for 1) ability to switch from benzodiazepine to alpidem and 2) ability to taper off alpidem | Negative |
| 102 | Lader et al. | 1987 | RCT | 24 | 39 | Buspirone and GDR vs. placebo and GDR | Any benzodiazepine | Enablement | Benzodiazepine discontinuation, withdrawal symptoms | Negative |
| 103 | Lader et al. | 2009 | Narrative review | NA | NA | Patient education, letter, GDR, pharmacological therapy | NA | NA | NA | NA |
| 104 | Lähteenmäki et al. | 2013 | RCT | 92 | 67 | Melatonin vs. placebo | Any benzodiazepine or Z-drug | Enablement, education, training | Benzodiazepine discontinuation or dose reduction (after 1 month and after 6 months) | Negative |
| 105 | Landry et al. | 1992 | Narrative review | NA | NA | GDR, pharmacological therapies, long-acting benzodiazepine substitution | NA | NA | NA | NA |
| 106 | Lemoine et al. | 1995 | RCT | 394 | Not specified | GDR vs. treatment continuation | Zopiclone, zolpidem | Enablement | Withdrawal symptoms | Negative |
| 107 | Lemoine et al. | 2006 | RCT | 160 | 48 | Cyamemazine substitution and GDR vs. bromazepam substitution and GDR | Any benzodiazepine | Enablement | Rebound anxiety, withdrawal symptoms, benzodiazepine use status | Positive |
| 108 | Lopez-Peig et al. | 2012 | Before-after study | 51 | 70 | Nurse-led benzodiazepine reduction program | Any benzodiazepine or Z-drug | Education, training, environmental restructuring, enablement | Benzodiazepine use status at 6 and 12 months, quality of life, sleep measures | Positive |
| 109 | Mackinnon et al. | 1982 | Narrative review | NA | NA | GDR, pharmacological substitution | NA | NA | NA | NA |
| 110 | Mant | 2002 | Narrative review | NA | NA | Letter (brief interventions), pharmacist medication reviews | NA | NA | NA | NA |
| 111 | Marriott et al. | 1993 | Narrative review | NA | NA | GDR, self-help groups, pharmacological therapies | NA | NA | NA | NA |
| **Ref** | **Source** | **Year** | **Article type** | **Sample size** | **Mean age** | **Deprescribing interventions studied or discussed** | **Medication discontinued** | **Intervention functions** | **Key study outcomes** | **Direction of effect** |
| 112 | Mercier-Guyon et al. | 2004 | RCT | 81 | 41 | GDR and captodiamine vs. GDR and placebo | Any benzodiazepine | Enablement, education | Withdrawal symptoms, anxiety symptoms, drowsiness, physical/mental performance, sleep quality, benzodiazepine use status | Positive |
| 113 | Michelini et al. | 1996 | Narrative review | NA | NA | GDR, CBT, follow-up, long-acting benzodiazepine substitution, flexible schedule, pharmacological therapies | NA | NA | NA | NA |
| 114 | Morin et al. | 2004 | RCT | 76 | 63 | GDR alone vs. CBT for insomnia vs. GDR and CBT for insomnia | Any benzodiazepine | Education, training, enablement | Benzodiazepine use status, sleep parameters, psychiatric rating scales (anxiety, depression), withdrawal symptoms, adherence | Positive |
| 115 | Morin et al. | 1995 | Non-comparative study | 5 | 63 | Effect of CBT and GDR on hypnotic use | Any benzodiazepine | Education, Training, Enablement | Sleep/wake patterns, type/dosage of benzodiazepine, benzodiazepine discontinuation | Positive |
| 116 | Morton et al. | 1995 | RCT | 26 | 46 | Buspirone and GDR vs. placebo and GDR | Any benzodiazepine | Enablement | Completion of withdrawal program, anxiety, mood, withdrawal symptoms, adverse effects | Negative |
| 117 | Mugunthan et al. | 2011 | Systematic review and meta-analysis | 615 | Not specified | Minimal interventions | Any benzodiazepine | Education, persuasion | Benzodiazepine discontinuation or dose reduction | Positive |
| 118 | Murphy et al. | 1991 | RCT | 68 | 45 | GDR with diazepam vs. GDR with lorazepam vs. GDR with bromazepam | Any benzodiazepine | Enablement | Withdrawal symptoms, psychiatric symptoms | Negative |
| 119 | Nakao et al. | 2006 | RCT | 66 | 59 | Paroxetine and GDR vs. GDR alone vs. no intervention | Any benzodiazepine | Enablement | Benzodiazepine discontinuation, withdrawal symptoms, anxiety symptoms | Positive |
| 120 | Nardi et al. | 2010 | Before-after study | 73 | 49 | Efficacy of GDR | Clonazepam | Enablement | Withdrawal symptoms, panic disorder symptoms, benzodiazepine discontinuation | Positive |
| 121 | Nathan et al. | 1986 | RCT | 7 | Not specified | Intensive psychotherapy vs. stress management | Any benzodiazepine | Education, training, enablement | Withdrawal symptoms, benzodiazepine use status | Unclear |
| 122 | National Prescribing Service, Australia | 2010 | Narrative review | NA | NA | Brief interventions, GDR, follow-up, education, psychological therapies | NA | NA | NA | NA |
| 123 | National Prescribing Service, Australia | 1999 | Narrative review | NA | NA | Education, monitoring, long-acting benzodiazepine substitution, GDR, flexibility | NA | NA | NA | NA |
| 124 | NHS, United Kingdom | 2013 | Guideline | NA | NA | Letter, education, GDR, long-acting benzodiazepine substitution, flexibility | NA | NA | NA | NA |
| 125 | Not specified | 2004 | Narrative review | NA | NA | GDR, psychological therapy, support, long-acting benzodiazepine substitution | NA | NA | NA | NA |
| 126 | NOUGG, Canada | 2010 | Guideline | NA | NA | GDR, follow-up, long-acting benzodiazepine substitution | NA | NA | NA | NA |
| **Ref** | **Source** | **Year** | **Article type** | **Sample size** | **Mean age** | **Deprescribing interventions studied or discussed** | **Medication discontinued** | **Intervention functions** | **Key study outcomes** | **Direction of effect** |
| 127 | Noyes et al. | 1988 | Narrative review | NA | NA | Patient education, GDR, long-acting benzodiazepine substitution, role of pharmacological agents, follow-up, support | NA | NA | NA | NA |
| 128 | NSW Department of Health, Australia | 2008 | Guideline | NA | NA | Stabilization, long-acting benzodiazepine substitution, GDR, flexibility, patient education | NA | NA | NA | NA |
| 129 | O'Connor et al. | 2008 | RCT | 86 | 48 | Group CBT and GDR vs. group support and GDR vs. usual care | Any benzodiazepine | Education, training, environmental restructuring, enablement | Withdrawal symptoms, psychological measures, benzodiazepine discontinuation | Unclear |
| 130 | Onyett | 1989 | Narrative review | NA | NA | Pharmacological therapies, follow-up, long-acting benzodiazepine substitution, psychological therapies | NA | NA | NA | NA |
| 131 | Onyett et al. | 1988 | RCT | 18 | 56 | Group training vs. individual appointment | Any benzodiazepine | Enablement, education, training | Benzodiazepine dose, anxiety and depression symptoms, withdrawal symptoms | Negative |
| 132 | Otto et al. | 2002 | Narrative review | NA | NA | CBT, GDR | NA | NA | NA | NA |
| 133 | Otto et al. | 2010 | RCT | 47 | 40 | Conservative GDR vs. GDR and individual relaxation treatment vs. GDR and single exposure-based CBT | Alprazolam, clonazepam | Enablement, education, training | Benzodiazepine use status, withdrawal symptoms, anxiety/mood, panic attack frequency | Positive |
| 134 | Otto et al. | 1992 | Narrative review | NA | NA | CBT, GDR | NA | NA | NA | NA |
| 135 | Otto et al. | 1993 | RCT | 33 | 38 | GDR vs. GDR and group CBT | Alprazolam, clonazepam | Enablement, education, training | Completion of scheduled taper, anxiety symptoms, withdrawal symptoms, number of panic attacks | Positive |
| 136 | Oude Voshaar et al. | 2006 | Systematic review and meta-analysis | 2,398 | 58 | Minimal intervention vs. systematic discontinuation vs. psychotherapy vs. pharmacologic augmentation | Any benzodiazepine | Enablement, education, training | Benzodiazepine discontinuation | Positive |
| 137 | Oude Voshaar et al. | 2003 | RCT | 180 | 63 | CBT and GDR vs. GDR alone vs. routine care | Any benzodiazepine | Education, training, enablement | Benzodiazepine discontinuation or dose reduction, withdrawal symptoms | Negative |
| 138 | Parr et al. | 2008 | Systematic review and meta-analysis | 21,912 | 52 | Routine care vs. brief interventions, GDR, and psychological therapy GDR alone vs. GDR and psychological therapy and substitutive pharmacotherapy | Any benzodiazepine or Z-drug | Enablement, education, training | Benzodiazepine discontinuation | Positive |
| 139 | Pat-Horenczyk et al. | 1998 | RCT | 24 | 49 | Benzodiazepine switch to zopiclone followed by GDR vs. benzodiazepine continuation and GDR | Flunitrazepam | Enablement, education, training | Sleep and psychological parameters, withdrawal symptoms, benzodiazepine use status | Positive |
| 140 | Pecknold | 1993 | Narrative review | NA | NA | GDR | NA | NA | NA | NA |
| 141 | Peles et al. | 2007 | RCT | 80 | 43 | Melatonin vs. placebo | Any benzodiazepine | Enablement | Sleep measures, benzodiazepine discontinuation, benzodiazepine use relapse | Negative |
| **Ref** | **Source** | **Year** | **Article type** | **Sample size** | **Mean age** | **Deprescribing interventions studied or discussed** | **Medication discontinued** | **Intervention functions** | **Key study outcomes** | **Direction of effect** |
| 142 | Petrovic et al. | 2003 | Narrative review | NA | NA | Psychological therapies, GDR, management of underlying condition | NA | NA | NA | NA |
| 143 | Poyares et al. | 2002 | RCT | 19 | 43 | Valarian vs. placebo | Any benzodiazepine | Enablement | Sleep measures, benzodiazepine use status | Unclear |
| 144 | Raju et al. | 2005 | Before-after study | 158 | 49 | Effect of discontinuation program | Any benzodiazepine or Z-drug | Education, enablement | Benzodiazepine discontinuation | Unclear |
| 145 | Rickels et al. | 2000 | Nonrandomized controlled trial | 75 | 48 | Buspirone vs. imipramine vs. placebo | Diazepam, lorazepam, alprazolam | Enablement | Benzodiazepine use status at 12 weeks and 12 months, withdrawal symptoms | Positive |
| 146 | Rickels et al. | 1999 | RCT | 78 | 47 | Trazodone vs. valproate vs. placebo | Diazepam, lorazepam, alprazolam | Enablement | Withdrawal symptoms, benzodiazepine use status | Negative |
| 147 | Rickels et al. | 1999 | Narrative review | NA | NA | Brief counselling, CBT, GDR, pharmacological therapies, treat underlying conditions, flexibile | NA | NA | NA | NA |
| 148 | Romach et al. | 1998 | RCT | 108 | 47 | Ondansetron vs. placebo | Alprazolam, lorazepam | Enablement, education, training | Benzodiazepine discontinuation or dose reduction, withdrawal symptoms, anxiety symptoms | Negative |
| 149 | Roth | 1989 | Narrative review | NA | NA | GDR, flexible schedule, long-acting benzodiazepine substitution, psychological support, pharmacological therapies | NA | NA | NA | NA |
| 150 | Roy-Bryne et al. | 1993 | Narrative review | NA | NA | Psychological therapies, pharmacological therapies | NA | NA | NA | NA |
| 151 | Roy-Byrne et al. | 1990 | Narrative review | NA | NA | GDR, pharmacological therapies | NA | NA | NA | NA |
| 152 | Roy-Byrne et al. | 1988 | Narrative review | NA | NA | Withdrawal symptoms, long-acting benzodiazepine substitution | NA | NA | NA | NA |
| 153 | Rynn et al. | 2003 | Nonrandomized controlled trial | 40 | Not specified | Placebo vs. buspirone vs. imipramine | Diazepam, lorazepam, alprazolam | Enablement | Psychiatric rating scales (anxiety, depression), benzodiazepine use status, withdrawal symptoms | Negative |
| 154 | Sanchez-Craig et al. | 1987 | RCT | 42 | 41 | CBT and abrupt stop vs. CBT and GDR | Any benzodiazepine | Enablement, education, training | Benzodiazepine discontinuation, withdrawal symptoms | Negative |
| 155 | Saul et al. | 1989 | RCT | 121 | 44 | Atenolol and GDR vs. placebo and GDR | Any benzodiazepine | Enablement | Withdrawal symptoms, anxiety symptoms, benzodiazepine use status | Negative |
| 156 | Schweizer | 1998 | Narrative review | NA | NA | Long-acting benzodiazepine substitution | NA | NA | NA | NA |
| 157 | Schweizer et al. | 1986 | Nonrandomized controlled trial | 15 | 43 | Abrupt discontinuation and replacement with buspirone vs. gradual discontinuation and replacement with buspirone | Any benzodiazepine | Enablement | Withdrawal symptoms, benzodiazepine use status, anxiety symptoms | Negative |
| 158 | Schweizer et al. | 1991 | RCT | 55 | 47 | Carbamazepine and GDR vs. placebo and GDR | Diazepam, lorazepam, alprazolam | Enablement | Withdrawal symptoms, benzodiazepine use status, anxiety and depression symptoms | Negative |
| **Ref** | **Source** | **Year** | **Article type** | **Sample size** | **Mean age** | **Deprescribing interventions studied or discussed** | **Medication discontinued** | **Intervention functions** | **Key study outcomes** | **Direction of effect** |
| 159 | Schweizer et al. | 1990 | Nonrandomized controlled trial | 63 | 47 | GDR of short half-life benzodiazepine vs. GDR of long half-life benzodiazepine | Any benzodiazepine | Enablement | Withdrawal symptoms, benzodiazepine use status, anxiety and depression symptoms | Negative |
| 160 | Schwiezer et al. | 1995 | RCT | 43 | Not specified | Progesterone and GDR vs. placebo and GDR | Diazepam, lorazepam, alprazolam | Enablement | Withdrawal symptoms, benzodiazepine use status, anxiety and depression symptoms | Negative |
| 161 | Shapiro et al. | 1995 | RCT | 134 | 50 | Zopiclone for benzodiazepine discontinuation | Any benzodiazepine | Enablement | Benzodiazepine use status, sleep measures, withdrawal symptoms, adverse effects | Positive |
| 162 | Sloan et al. | 2013 | Narrative review | NA | NA | GDR, treat underlying condition, education, follow-up, long-acting benzodiazepine substitution, flexible approach, restricted dispensing | NA | NA | NA | NA |
| 163 | Smith et al. | 2010 | Narrative review | NA | NA | Multi-faceted approach, support groups for patients | NA | NA | NA | NA |
| 164 | Spiegel et al. | 1999 | Narrative review | NA | NA | Education, flexible GDR, collaboration with patient, managing underlying conditions, CBT | NA | NA | NA | NA |
| 165 | Stewart et al. | 2007 | Nonrandomized controlled trial | 8,170 | 65 | Letter encouraging benzodiazepine cessation vs. no intervention | Any benzodiazepine or Z-drug | Education, training, environmental restructuring | Change in benzodiazepine prescribing by physician | Positive |
| 8 | Tannenbaum et al. | 2014 | RCT | 303 | 75 | Direct to consumer educational intervention vs. usual care | Any benzodiazepine | Education, persuasion, modelling | Benzodiazepine discontinuation or dose reduction | Positive |
| 166 | Taylor et al. | 2010 | RCT | 46 | 54 | Sleep hygiene education, sleep restriction, and hypnotic withdrawal vs. sleep hygiene education alone | Any benzodiazepine or Z-drug | Enablement, education, training | Sleep measures, benzodiazepine or Z-drug use status | Positive |
| 167 | Therapeutics Letter | 1997 | Narrative review | NA | NA | Education, long-acting benzodiazepine substitution, GDR, follow-up | NA | NA | NA | NA |
| 168 | Thirtala et al. | 2013 | Narrative review | NA | NA | Education, psychotherapy, treat underlying conditions, GDR, long-acting benzodiazepine substitution | NA | NA | NA | NA |
| 169 | Tiller et al. | 2010 | Narrative review | NA | NA | Education, GDR, psychological therapies, brief interventions, flexibility, long-acting benzodiazepine substitution, follow-up, engage family and carers | NA | NA | NA | NA |
| 170 | Tyrer et al. | 1996 | RCT | 87 | Not specified | Dothiepin and GDR vs. placebo and GDR | Any benzodiazepine | Enablement | Benzodiazepine discontinuation, withdrawal symptoms, anxiety and depression symptoms, adverse effects | Unclear |
| 171 | Tyrer et al. | 1984 | Narrative review | NA | NA | GDR, long-acting benzodiazepine substitution, pharmacologic therapies | NA | NA | NA | NA |
| 172 | Udelman et al. | 1990 | RCT | 72 | 42 | Buspirone and GDR vs. placebo and GDR | Alprazolam | Enablement | Alprazolam discontinuation, withdrawal symptoms, anxiety symptoms | Negative |
| **Ref** | **Source** | **Year** | **Article type** | **Sample size** | **Mean age** | **Deprescribing interventions studied or discussed** | **Medication discontinued** | **Intervention functions** | **Key study outcomes** | **Direction of effect** |
| 173 | van de Steeg-van Gompel et al. | 2009 | RCT | 19,398 | 65 | Pharmacists receiving training vs. pharmacists receiving manual only for implementation of a benzodiazepine discontinuation letter | Any benzodiazepine or Z-drug | Education | Physician and pharmacist participation in intervention, benzodiazepine discontinuation or dose reduction | Unclear |
| 174 | Vicens et al. | 2006 | RCT | 139 | 59 | Benzodiazepine withdrawal advice and biweekly follow-up vs. routine care | Any benzodiazepine | Education, enablement | Benzodiazepine discontinuation or dose reduction | Negative |
| 175 | Vicens et al. | 2014 | RCT | 532 | 64 | Clinic visit follow-up vs. written letter and follow-up vs. usual care | Any benzodiazepine or Z-drug | Education, enablement | Benzodiazepine discontinuation, anxiety and depression symptoms, sleep quality, alcohol consumption, withdrawal symptoms | Positive |
| 176 | Vissers et al. | 2007 | RCT | 38 | Not specified | Melatonin and GDR vs. placebo and GDR | Any benzodiazepine | Enablement | Benzodiazepine discontinuation | Negative |
| 177 | Vorma et al. | 2002 | RCT | 76 | 40 | GDR and CBT vs. routine care | Any benzodiazepine or Z-drug | Enablement, education, training | Taper completion, benzodiazepine discontinuation or dose reduction | Negative |
| 178 | Woodward | 2000 | Narrative review | NA | NA | Education, follow-up, long-acting benzodiazepine substitution, flexible GDR, sleep hygiene | NA | NA | NA | NA |
| 179 | Woodward | 1999 | Narrative review | NA | NA | GDR, long-acting benzodiazepine substitution, relaxation training | NA | NA | NA | NA |
| 180 | Zee | 2011 | RCT | 135 | 49 | Placebo and GDR vs. ramelteon and GDR | Zolpidem | Enablement | Zolpidem discontinuation | Negative |
